# Supplementary material for: Genetic LGALS1 Variants Are Associated with Heterogeneity in Galectin-1 Serum Levels in Patients with Early Arthritis
Source: Int J Mol Sci. 2022 Jun 28;23(13):7181. doi: 10.3390/ijms23137181 (PMC9266574; doi:10.3390/ijms23137181)
Supplement: Supplementary file 1 [file ijms-23-07181-s001.zip › ijms-1775262-supplementary.pdf]

Genetic *LGALS1* variants are associated with heterogeneity in Galectin-1 serum levels in patients with early arthritis.

Ana Triguero-Martínez[1] BS, ana6n92@gmail.com

Emilia Roy-Vallejo[2] MD, eroyvallejo@gmail.com

Nuria Montes[1] PhD, nuria.montes.casado@gmail.com

Hortensia de la Fuente[3] PhD, hortensiadelafuente@gmail.com

Ana María Ortiz[1] MD, PhD, lanult@yahoo.es

Santos Castañeda[1] MD,PhD, scastas@gmail.com

Isidoro González-Alvaro[1]*MD, PhD, isidoro.ga@ser.es Phone: 34-915202438. Fax: 34-915202374.

Amalia Lamana[4]*PhD, amaliala@ucm.es Phone: 34- 91 394 49 82

[1]Rheumatology Department, Hospital Universitario La Princesa, Instituto de Investigación Sanitaria La Princesa (IIS-IP), Madrid. Spain.

[2]Internal Medicine Department, Hospital Universitario La Princesa, Instituto de Investigación Sanitaria La Princesa (IIS-IP), Madrid. Spain

[3]Immunology Department, Hospital Universitario La Princesa, Instituto de Investigación Sanitaria La Princesa (IIS-IP), Madrid. Spain.

[4]Cell Biology Department, Facultad de Biología, Universidad Complutense de Madrid, Madrid, Spain.

* These authors share corresponding authorship

SUPPLEMENTARY MATERIAL

SUPPLEMENTARY FIGURES

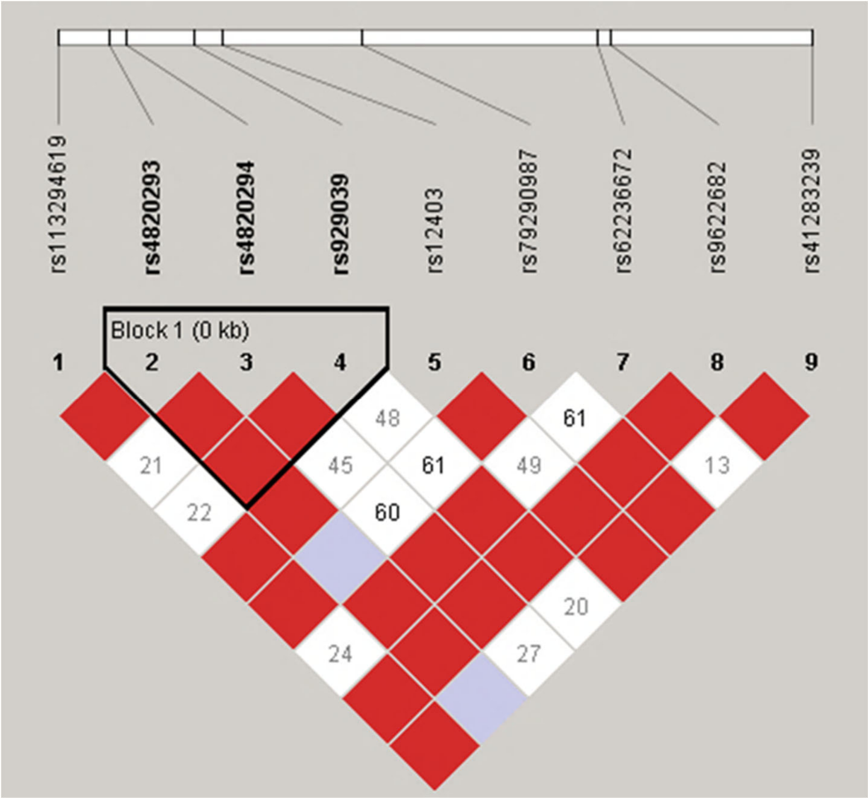

Figure S1. Linkage disequilibrium (LD) plot of the single nucleotide polymorphisms (SNPs) studied in the *LGALS1* gene and adjacent regions.

LD plot of common variants (minor allele frequency threshold 0.01) in the *LGALS1* region based on sequencing data of 53 early arthritis patients from PEARL study. Plot shows D' (normalized linkage disequilibrium measure or D) values. LOD was defined as $\log_{10}(L1/L0)$, where L1 = likelihood of the data under linkage disequilibrium, and L0 = likelihood of the data under linkage equilibrium. D' was calculated as follows: D' = (D) divided by the theoretical maximum for the observed allele frequencies. Red blocks, D' ≤1.0, with logarithm of odds (LOD) score ≥2.0; white blocks, D' <1.0 with LOD <2.0; blue blocks, D' =1.0 with LOD <2.0. Numbers in blocks denote D' values. The genomic organization is described above the LD plot; the *LGALS1* gene was located

between rs12403 and rs41283239 (Chr22: 37675700..37679827). LD plot of genotyping variants in early arthritis population from PEARL study.

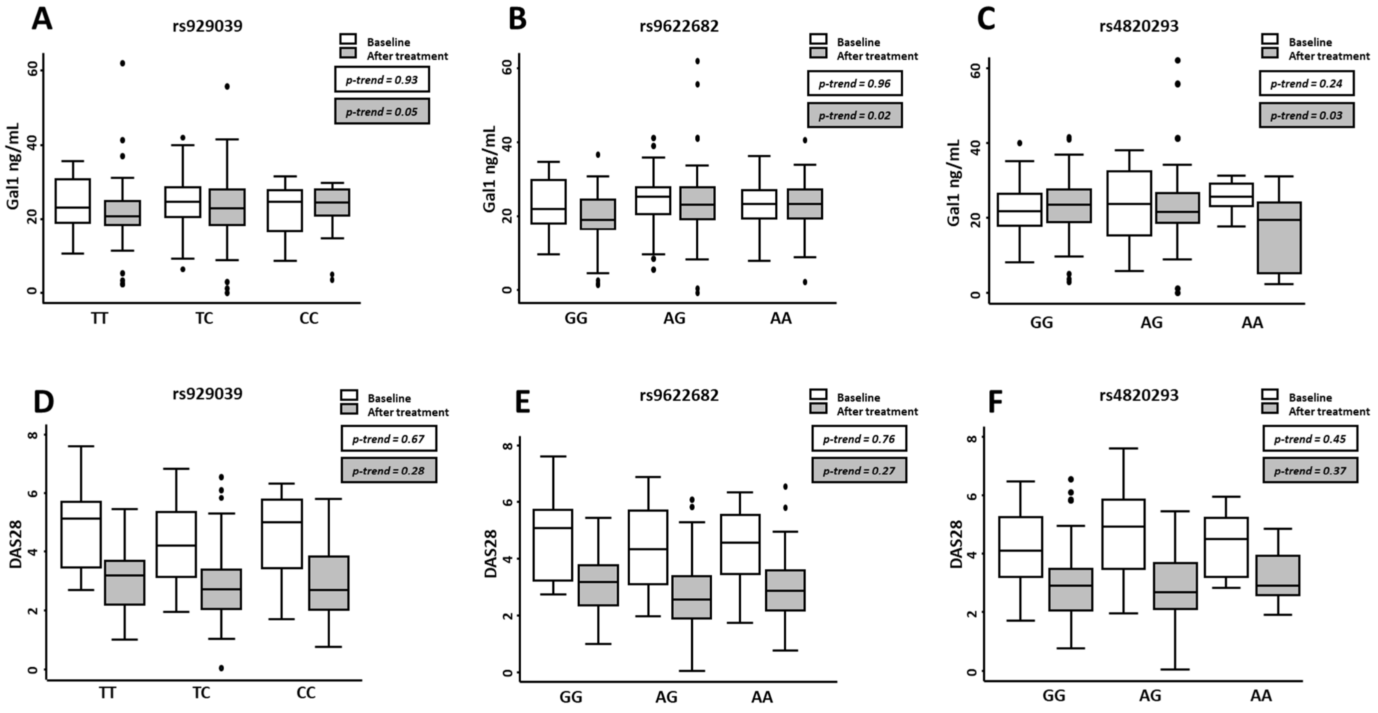

Figure S2. Effect of the *LGALS1* SNPs rs929039, rs9622682 and rs4820293 in galectin 1 (Gal1) serum levels and disease activity along the follow-up.

Determination of Gal1 serum levels by ELISA in early arthritis patients from PEARL study according to the different genotypes for the genetic variants rs929039 (A), rs9622682 (B), rs4820293 (C) in baseline visit (white boxes) and after treatment prescription (grey boxes). Relationship between disease activity assessed by DAS28 according to the different genotypes for the genetic variants rs929039 (D), rs9622682 (E), rs4820293 (F) in baseline visit (white boxes) and after treatment prescription (grey boxes). Data are shown as interquartile range (p75 upper edge of box, p25 lower edge, p50 midline) as well as the p95 (line above box) and p5 (line below). Dots represent outliers. Statistical significance for the trend of Gal1 across the different genotypes in patients was determined with Cuzick's non-parametric test. Significance threshold was set at p-trend<0.05.

SUPPLEMENTARY TABLES

Table S1. Sequences of the primers used for sequencing *LGALS1* gene and length of generated amplicons.

Amplicon	Length (bp)	Primer sequence forward (5´-M13-PRIMER-3´)	Primer sequence reverse (5´-M13-PRIMER-3´)
Amplicon 1	463	5´AGGAGATGTTAAGAGAGCAGACAGGGT-3´	5´TAGTCAAAGCGCCCTGGCCT-3´
Amplicon 2	681	5´ACAGGGTGCACAGAGCCAGG-3´	5´GGGCGGAGTCCGTGGAATTT -3´
Amplicon 3	721	5´GCCATCTTCTCTGGGCACCC-3´	5´GCAGAATGTCAACCCTGGGC-3´
Amplicon 4	622	5´TCCCTCAAGGCCTTCTCTGCA-3´	5´TAGAGAGGAGACCCTGAAAAACAC-3´
Amplicon 5	504	5´TCAAACCAGTTAACTCTTGCCC-3´	5´AAAGGATTGAGCTGTAATTTCCAG-3´
Amplicon 6	713	5´CTCCATTCAGTGTGACGGTG-3´	5´TACAGATGACGAGGCTGCAG-3´
Amplicon 7	730	5´CCGCTCCCTCCCTGCTGTAG-3´	5´GTGAAACCCCGTCTCTACTAAAAG-3´
Amplicon 8	727	5´ATGCACTAATTTCATTTCAACCAA-3´	5´TCCCATGAACGCACCTTGCT-3´
Amplicon 9	570	5´TGACCAAACGGGCTAGGATGC-3´	5´GGGCAGACACTGGCCATCAC-3´
Amplicon 10	492	5´AGAGACCTTGGCCCTGCCTG-3´	5´CTCAGGTGATCCACCCACTT-3´
Amplicon 11	770	5´GTGATGGCCAGTGTCTGCCC-3´	5´CGTTCCTCATGTGGCCCTGA-3´
Amplicon 12	728	5´AAGTGGGTGGATCACCTGAG-3´	5´ACCCGTGGACAAAGCGAATG-3´

BP:base pairs

Table S2. Genetic variants of *LGALS1* gene.

dbSNP rs#	Amplicon	Variant	Region	Localitation (GRCh38.p2)	MAF (sequencing)	MAF (CEU population)	Genotyping (n)	MAF (Genotyping)
rs113294619	1	T>C	Promoter	37674552	0.11	0.02		
rs4820293	1,2	A>G	Promoter	37674914	0.44	0.38	503	0.31
rs4820294	2	G>A	Promoter	37675036	0.31	0.34		
rs929039	2,3	T>C	Promoter	37675504	0.31	0.3	523	0.39
rs12403	3	A>G	Exon1	37675700	0.13	0.05		
rs3985986	4	C>G	Intron1	37676225	0.19	-		
rs79290987	5,6	C>G	Intron6	37676678	0.11	0.01		
rs12167604	8	T>C	Intron3	37678135	0.33	0.46		
rs62236672	8,9	C>T	Intron5	37678336	0.33	0.34		
rs9622682	9	G>A	Intron6	37678427	0.43	0.45	521	0.49
rs41283239	12	G>A	Intron4	37679827	0.22	0.03		

MAF: minor allele frequency; CEU: Central European reference population; n: number

Table S3. Relationship between IL-6 (pg/ml) serum levels and *LGALS1 and IL-6R* genetic variants.

	LGALS1 (rs929039)		*LGALS1* (rs9622682)		*LGALS1* (rs4820293)	
	**β Coeff. (95% CI)**	**p value**	**β Coeff. (95% CI)**	**p-value**	**β Coeff. (95% CI)**	**p value**
**DAS28**						
Remission	Reference	–	Reference	–	Reference	–
Low DA	0.20 (0.06 to 0.33)	0.004	0.16 (0.02 to 0.29)	0.02	0.23 (0.09 to 0.38)	0.01
Moderate DA	0.66 (0.50 to 0.82)	<0.001	0.65 (0.49 to 0.81)	<0.001	0.74 (0.57 to 0.91)	<0.001
High DA	1.31 (0.93 to 1.69)	<0.001	1.31 (0.93 to 1.70)	<0.001	1.27 (0.90 to 1.65)	<0.001
**Methotrexate dose (mg)**	−0.01 (−0.02 to −0.007)	<0.001	−0.01 (−0.02 to −0.006)	<0.001	−0.01 (−0.02 to −0.005)	0.001
***IL-6R* (rs2228145)**						
AA	Reference		Reference	–	Reference	–
AC	0.35 (0.16 to 0.55)	<0.001	0.28 (0.08 to 0.48)	0.004	0.32 (0.12 to 0.51)	0.001
CC	0.25 (−0.01 to 0.51)	0.06	0.25 (−0.19 to 0.52)	0.069	0.27 (−0.015 to 0.55)	0.15
***LGALS1* (rs929039)**						
TT	Reference	–				
TC	−0.38 (−0.60 to −0.15)	0.001				
CC	−0.36 (−0.66 to −0.69)	0.01				
***LGALS1* (rs9622682)**						
GG			Reference	–		
GA			−0.40 (− 0.67 to − 0.14)	0.003		
AA			−0.48 (−0.76 to −0.20)	0.001		
***LGALS1* (rs4820293)**						
GG					Reference	–
GA					0.09 (− 0.09 to − 0.27)	0.33
AA					0.58 (0.15 to 1)	0.008

DAS28: disease activity score estimated with 28 joint count; DA: disease activity; IL-6R: IL-6 receptor, Coeff: coefficient; CI: confidence interval.

Table S4. Relationship between IL-6 (pg/ml) serum levels and *LGALS1 and IL-6* genetic variants.

	LGALS1 (rs929039)		*LGALS1* (rs9622682)		*LGALS1* (rs4820293)	
	**β Coeff. (95% CI)**	**p value**	**β Coeff. (95% CI)**	**p value**	**β Coeff. (95% CI)**	**p value**
**DAS28**						
Remission	Reference	–	Reference	–	Reference	–
Low DA	0.21 (0.06 to 0.35)	0.004	0.18 (0.04 to 0.33)	0.009	0.25 (0.1 to 0.41)	0.001
Moderate DA	0.67 (0.50 to 0.84)	<0.001	0.68 (0.52 to 0.85)	<0.001	0.73(0.55 to 0.90	<0.001
High DA	1.29 (0.91 to 1.68)	<0.001	1.34 (0.94 to 1.73)	<0.001	1.25 (0.86 to 1.63)	<0.001
**Methotrexate dose (mg)**	−0.01 (−0.02 to −0.004)	0.003	−0.01 (−0.01 to −0.002)	0.008	−0.01 (−0.02 to −0.002)	0.009
***IL-6* (rs1800795)**						
GG	Reference		Reference	–	Reference	–
GC	−0.14 (−0.34 to −0.04)	0.12	−0.14 (−0.33 to −0.04)	0.14	−0.15 (−0.35 to −0.05)	0.14
CC	−0.03 (−0.43 to −0.37)	0.87	−0.06 (−0.44 to −0.30)	0.72	−0.09 (−0.48 to −0.30)	0.64
***LGALS1* (rs929039)**						
TT	Reference	–				
TC	−0.39 (−0.63 to −0.14)	0.002				
CC	−0.50 (−0.80 to −0.20)	0.001				
***LGALS1* (rs9622682)**						
GG			Reference	–		
GA			−0.44 (− 0.72 to − 0.16)	0.002		
AA			−0.58 (−0.88 to −0.28)	<0.001		
***LGALS1* (rs4820293)**						
GG					Reference	–
GA					0.1 (− 0.09 to − 0.30)	0.29
AA					0.63 (0.16 to 1.1)	0.008

DAS28: disease activity score estimated with 28 joint count; DA: disease activity; Coeff: coefficient; CI: confidence interval.
